# The Story of the Insane from Year to Year

**Published:** 1890-09-27

**Authors:** 


### The Story of the Insane from Year to Year.

[Annual Reports, Pamphlets, &c., should be addressed to The Editor, The Lodge, Porchester Square, London, W.]

### SUSSEX COUNTY ASYLUM.

This is the thirty-first annual report, so a new generation has begun for the asylum. It has been a little crowded. The Hastings patients have been turned out, and 85 other cases have been sent to the Northamptonshire Asylum. It does not appear what steps the visitors intend taking to remedy this want of room, but manifestly something must be done ere long. There were 820 patients in residence at the close of the year. The recoveries stand at 27·9 per cent.; the deaths at 11·6. Forty-six of the deaths were owing to brain diseases, and 17 to phthisis. The new Lunacy Act seems to please no one except the private asylum proprietors. Dr. Saunders winds up his criticism on it as follows:—"It will certainly throw a vast amount of extra work on the medical superintendent, for very inadequate results and merely to pander to a popular outcry, for no case of improper detention has ever been proved to have occurred in any public asylum in this country." With regard to the 40th section of the new Act, which provides for the maintenance of pauper lunatics taken charge of by their friends, we may point out that the old clause is not repealed, and it will probably be used in preference to the new one. The Commissioners say "that the late committee have handed it (the asylum) over to their successors in a highly creditable state."

### THE WORCESTER COUNTY ASYLUM.

During the year 1889 the average number resident was 876, and the total number in the asylum at the close of the year was 897, showing an increase of 58. The percentage of recoveries stands at 29·1 on the admissions, and the deaths at 7·9 on the average number resident. The total number of admissions was 199, and Dr. Cooke shows that 120 of them were hopelessly incurable on admission. We commend the table given at page 24 of the report to those people who are clamouring for an acute hospital, wherein they would cure 60 per cent. of the cases. We learn from the Commissioners' report that this asylum is not supplied with mess rooms for the attendants, in which want it must stand almost alone among county asylums. The staff of attendants would seem to be numerically weak, while the medical staff is, if anything, unnecessarily strong. The maintenance rate is only 7s. per head per week, a sum which we believe to be too low. The visitors record their complete satisfaction with the manner in which the asylum is conducted by Dr. Cooke. The Medical Superintendent's report should follow that of the Committee of Visitors.

### SOMERSET COUNTY ASYLUM.

This asylum now contains 864 patients, showing an increase of 60 on the number for the previous year. The male side of the building is completely full, and the female side is almost so. The visitors came to the sensible conclusion that it would be a mistake to enlarge the old asylum, or to build a separate asylum for chronic lunatics, and they decided to build an entirely new asylum in the western part of the county. Dr. Wade's report is a very able one. He discusses the various suggestions which have from time to time been made as to the best means of dealing with the insane. "Speaking from a purely medical point of view," he says, "I should deeply regret the separation of the different classes of the insane into different asylums. . . . If the separate provision were to be only for the quiet and so-called harmless, leaving the dangerous chronic patients for treatment here, it would be found that the number who could be removed would be few."

### BROADMOOR ASYLUM, CROWTHORNE.

This is the asylum for criminal lunatics, and at the date of its last issued report it contained 618 patients, of whom 471 were men, and 147 women. Of these 509 are detained during her Majesty's pleasure, 106 are under sentence of penal servitude—that is, they are convicts—and three are under sentence for shorter periods. Of the total, 308 had committed homicide. Two men and three women were discharged to the care of relatives, upon warrants of conditional release, but three of these were readmitted owing to recurrence of the insanity. Nineteen men and four women were transferred to other asylums. There were only 19 deaths during the year. The most of these were owing to brain disease; and not one was caused by violence, suicide, or accident, a strong proof of Dr. Nicholson's admirable management. When speaking of restraint he says: "Few people who have not been engaged in it are able to estimate the wear and tear of nerve and heart which a tussle with a desperate lunatic means to the attendants, apart from the risk of positive injury either to patient or warders." Three patients escaped, one by means of a key, which he had made, one by climbing over the garden wall during a fog, and the third by leaping from some scaffolding. The two latter were speedily captured, but the first remains at large. The average cost per patient was £46 0s. 4d. per annum. The accommodation provided for criminal lunatics in England, and their treatment and surroundings, are, as will be seen from this report, very satisfactory; but there is another class, who have been named "lunatic criminals," for whom no proper provision exists. As a consequence they have to be sent to our county asylums, and very often indeed constitute the most troublesome cases with which the authorities have to deal. Scarcely a medical superintendent in England who has not pointed out this flaw in our Lunacy Acts; the remedy is simple enough, but it seems impossible to get it applied.

### WEST RIDING ASYLUM AT MENSTON.

This is the newest county asylum in England, and was opened on the 8th October, 1888. The asylum can accommodate 840 patients, and the administrative department is large enough for 1,340. At the end of the year there were 437 patients in residence. The recovery rate was 20 per cent. on admissions, and the deaths were 13·88 per cent. on the average number in residence. Many medical superintendents have had something to say about the hospital idea of treating the insane. Dr. Macdowall says, "Whether patients suffering from insanity would recover in greater numbers in a hospital situated in a large town, and provided with a staff of visiting physicians, than in an asylum in the country without such a staff, there is great reason to doubt." Very strong reason to doubt, we should say. Dr. Macdowall thinks, and we agree with him, that the nursing staff in asylums should be increased.

### WARNEFORD HOUSE, OXFORD.

This is one of the lunatic hospitals which really does a good deal of charitable work in relieving the trials which the insane of the middle-classes so often have to endure, and it therefore deserves the help of the rich. It contains about eighty patients. The average cost of maintenance was nearly £1 4s. per head per week, exclusive of repairs to the building, rates, and taxes. No fewer than 62 were maintained at charges less than £1 a-week—indeed, some of these paid less than 15s., and a few paid under 5s. Non-charitable cases are admitted at £2 2s. a-week. Neither epileptics, paralytics, nor idiots are admitted.

